# Developing a Female Sex Worker-Led Program to Improve the Uptake of Oral Pre-Exposure Prophylaxis in South Africa: An Intervention Mapping Study

**DOI:** 10.3390/ijerph22121862

**Published:** 2025-12-14

**Authors:** Nosipho Faith Makhakhe, Gift Khumalo

**Affiliations:** Centre for General Education, Durban University of Technology, Durban 4001, South Africa; nosiphom8@dut.ac.za

**Keywords:** female sex workers, intervention mapping, South Africa, pre-exposure prophylaxis

## Abstract

**Highlights:**

**What are the main findings?**

- This study demonstrates the systematic application of the Intervention Mapping framework to design a targeted strategy addressing the determinants of Pre-Exposure Prophylaxis (PrEP) uptake among FSWs.
- It highlights the efficacy of participatory approaches, illustrating how FSWs can be integrated into the research process not merely as beneficiaries but as active co-creators and decision-makers in the formulation of HIV prevention interventions.

**What are the implications of the main findings?**

- HIV prevention strategies must be multilevel in scope. Rather than relying solely on biomedical approaches, interventions must encompass the social, behavioral, and structural determinants of prevention. To ensure interventions are contextually appropriate, they must be predicated on a rigorous needs assessment.
- Meaningful engagement of the target population in both the design and implementation phases is essential to maximize intervention uptake and ensure long-term sustainability.

**Abstract:**

In 2016, the South African government approved free oral PrEP distribution among high-risk groups like female sex workers (FSWs) to reduce new HIV infections. Despite the availability, unique barriers exist that challenge PrEP persistence, including limited knowledge, side effects, stigma, and mobility that hinder adherence. As such, engaging FSWs in the design of an FSW-led intervention program is crucial to promote PrEP uptake, adherence, and retention. Processes of an intervention mapping approach were applied to design and develop the program in KwaZulu-Natal, South Africa. A needs analysis was completed through existing literature and through engagements with FSWs, FSW peer educators, and a healthcare provider. The working group, comprising eight FSW peer educators and a researcher, co-created the intervention following a six-step mapping process. A total of six meetings took place, during which intervention determinants, change objectives, theory-based methods, and the intervention program were discussed and formulated, as well as implementing partners and the evaluation plan identified. The program focuses on the development of agency, self-efficacy, and hope among FSWs and aims to destigmatize PrEP through positive messaging, equipping FSWs with the ability to differentiate PrEP from ARVs given to people living with HIV. Through role-playing, participants will practice discussing PrEP with their intimate partners and friends, receive suggestions on managing pill supply and side effects, and be equipped to become PrEP ambassadors. The introduction of PrEP as a pill for high-risk groups can be stigmatizing. Therefore, it is crucial to involve marginalized groups in the design and implementation of their interventions to foster acceptance and develop a sense of ownership, ensuring the programs’ sustainability.

## 1. Introduction

A significant number of studies have been conducted worldwide regarding the management and prevention of HIV. Research from various studies and clinical trials has proven the effectiveness and efficacy of HIV prevention programs, such as prevention from mother-to-child transmission, voluntary male medical circumcision, universal test and treat, and the provision of pre-/post-exposure prophylaxis (PrEP) [1]. The combination of these prevention programs has somewhat contributed to the relative decline of HIV infections worldwide [2]. However, despite the availability of a widening array of practical HIV prevention tools, there has been unequal progress in reducing new HIV infections, increasing access to treatment, and ending AIDS-related deaths, with many vulnerable people and populations left behind [2]. A persistent challenge is the lack of access to good and effective healthcare services for key populations. These groups, which consist of injecting drug users, sex workers, gay men and other men who have sex with men, transgender people, young women, and adolescent girls, face a disproportionately high HIV incidence and prevalence because of frequent exposure to HIV risk [3]. The Joint United Nations Programme on HIV/AIDS estimates that more than 60% of new HIV infections are among key populations and their sexual partners, highlighting the need to increase the focus of the HIV response on these groups [4]. In 2022, an estimated 8.45 million people in South Africa lived with HIV [5]. Statistics further confirm that HIV prevalence amongst female sex workers (FSWs) in South Africa is estimated to range between 39% and 72%, as recorded in three of South Africa’s major cities. There are various biomedical, behavioral, social, and structural challenges that FSWs in South Africa face, which exacerbate their exposure to HIV infections [6].

From a biomedical perspective, the cumulative exposure to HIV among FSWs is due to frequent engagement in sexual intercourse with multiple partners and irregular condom use. Behavioral and social factors known to influence irregular condom use stem from unequal power relations with clients and non-paying partners [7]. FSWs’ clients frequently include men who refuse to use condoms or who use force and intimidate FSWs to consent to sex without a condom, or who pay more money for such sex, or who threaten to take their business elsewhere if the sex worker requires the use of a condom [8]. Alcohol and drug use while working also influences FSWs’ capacity to negotiate and practice ‘safer’ sex [9]. The structural conditions that make it difficult for FSWs to practice safer sex are related to the criminalization of sex work, which exposes sex workers to violence and abuse that they cannot report. They also face harsh treatment from law enforcement officers who issue frequent unlawful arrests of FSWs; this has sex workers reluctant to report crimes committed against them by both clients and the police [10]. All these factors culminate in FSWs’ exposure to HIV risk.

It is thus essential to have targeted and tailored HIV prevention interventions and services for FSWs, which include condoms, antiretroviral treatment, and PrEP [11,12]. PrEP is an antiretroviral given to HIV-negative people to prevent them from acquiring HIV. The introduction of oral PrEP (tenofovir and emtricitabine) among high-risk groups where HIV incidence exceeds 2–3 in 100 persons, as per the WHO guidelines [12], was aimed at ensuring that those who are at high risk of contracting HIV have an additional prevention method to use concurrently with condoms [13]. The distribution of PrEP commenced on 1 June 2016, making South Africa the first country in sub-Saharan Africa to approve the distribution of PrEP among sex workers [14,15]. Since the launch of PrEP, research has shown that FSWs are willing to take up PrEP; however, several challenges affect uptake and adherence, including knowledge and beliefs about the efficacy of PrEP, forgetting to take PrEP, stigma, side effects, mobility issues, and missing clinic appointments. To mitigate these challenges, Ortblad and Oldenburg [16] recommend that combination prevention should factor in long-term peer support and community-based empowerment activities to encourage PrEP use. This paper outlines how intervention mapping was utilized as a framework and methodological process in the co-creation of a humanistic PrEP workshop program with FSWs, where the focus is not solely on pill taking or condom use, which is the hallmark of most HIV prevention interventions, to a holistic person-centered approach to prevention that seeks to explore and foster qualities such as agency which is an individual’s capacity to make their own choices and take positive action that shapes the trajectory of their own life [17]. Self-efficacy is defined as a person’s belief in their ability and skills to achieve a specific goal [18]. Hope is a motivational mindset focused on achieving goals. It is a way of thinking that requires the cognitive ability to set goals and the mental energy to continue on the chosen path even in the face of difficulty [19]. According to research, there is a need for multilevel interventions that not only focus on the biomedical aspects of HIV prevention but also incorporate the behavioral and structural factors that influence the ability of FSWs to engage in preventative behavior [20]. This paper details the process of how the Walk in Hope PrEP workshop program was designed as a behavioral intervention to address the barriers and facilitators of PrEP use among FSWs.

## 2. Materials and Methods

This study follows an intervention mapping (IM) approach for a PrEP workshop program with FSW. Specifically, the intervention mapping process was conducted in the coastal city of Durban, KwaZulu-Natal (KZN), one of South Africa’s nine provinces. KZN has a diverse population and significant public health and socio-economic challenges. Amongst the challenges are the high gender-based violence and sexual exploitation, the prevalence of HIV, with a higher prevalence in women aged 15–44 years (with 36.3%), and under-allocation of community health workers [21,22,23]. These challenges, especially those related to high HIV rates in the province and related factors, highlight the need for targeted prevention strategies and improved coverage of PrEP promotion.

The intervention mapping procedure followed Bartholomew et al.’s [24] step-by-step framework that guides planners during the intervention development process. The framework suggests six steps that provide a systematic integration of theory, empirical findings from the literature, as well as information collected from the target population [25]. These steps include (i) needs analysis; (ii) formulating the matrices of change objectives; (iii) developing theory-based methods and practical intervention strategies; (iv) integrating methods and practical strategies into an intervention workshop program; (v) demonstrating how the intervention will be implemented and adopted; and (vi) involving the development of a process and impact evaluation plan. The focus of the current manuscript is to outline the first four steps, as steps five and six are yet to be initiated.

### 2.1. Step 1: Needs Analysis

The needs analysis was a systematic investigation of the differences between the existing circumstances that prevail and the ideal circumstances. Intervention mapping guidelines stipulate that the needs analysis is a problem identification phase that should be based on the following principles: avoid blaming the victim, involve community participants, and examine the environmental causes of problems [24,26]. Moreover, during this first step, Bartholomew [24] notes that it is important to engage stakeholders who have a vested interest in the problem and possess insider information to provide a factual description of the problem as well as find relevant solutions to the challenges.

This step was achieved by conducting a needs analysis with 39 participants: 26 FSW (20 PrEP users and 6 non-PrEP users), 11 FSW peer educators, one healthcare provider, and one researcher. The inclusion criteria for study participants were women aged 18, who identify as sex workers and have been selling sex for a period of six months or more. The criteria for healthcare providers included healthcare providers or researchers working in sex work organizations or organizations that provide HIV prevention services to FSWs. The number of participants was determined by data saturation, where no new valuable insights emerged from the data collection [27]. Participants were recruited through snowball sampling [28] from two organizations—Sisonke, a national sex worker-led advocacy group, and TB HIV Care, a health service provider for key populations. The needs analysis examined FSWs’ experiences with PrEP, as well as investigating the barriers and facilitators of PrEP use among this group. This step also involved conducting a comprehensive literature search to gain a deeper understanding of the need.

The data for the needs analysis were analyzed thematically, which involved the six steps outlined by Braun and Clarke [29]. This process included sorting and coding the data into themes and categories by identifying and analyzing repeating patterns related to factors affecting PrEP uptake, adherence, and retention among FSWs. Specifically, the data were then grouped into personal and environmental factors.

### 2.2. Step 2: Identifying Objectives

The second step involved formulating change objectives, which involved the peer educators and the first author. The intention was that peer educators would also be the main intervention implementers at the initial stages of the program, to train other facilitators from the FSW community at a later stage as a way to encourage sustainability Bartholomew et al. [24] explain the process of this step as actions aimed at changing key behaviors that influence the desired outcomes and that it focuses on the expected outcomes, performances, and determinants for behavior and environment, constructing matrices for change objectives and creating a logic model of change. Regarding the expected outcomes, behavioral and environmental outcomes were identified as significant, as they specified specific changes necessary for the intervention to be effective, focusing on what behaviors needed to be accomplished by the FSWs and how the environment needed to change to support those behaviors. These outcomes were then linked to the performance and change objectives matrices related to the individual changes, interpersonal changes, and environmental changes. Each performance and change objective was linked to key determinants, which are the fundamental underlying factors that must be understood and often modified to achieve a specific goal or change a particular situation.

Thereafter, the logic model was developed, which is a visual representation of the systematic depiction of the intervention and its components [30]. It consists of the problem, the goal of the intervention, the inputs needed that will lead to the intervention activities, as well as outcomes, which can be classified as short-term, intermediate, and distal outcomes that will eventually contribute to the overall impact of the intervention. Logic models are essential because they ensure that program developers depict a systematic way of thinking and adjusting the program based on feasibility. In this case, the logic model was incorporated into Step 2 of the intervention mapping process to assist the stakeholders in having a shared understanding of how the program will be implemented, how change will be achieved through the intervention, and the various detailed activities that need to take place for that change to be realized, as outlined by Breuer et al. [31]. Each component of the logic model is essential and leads to another step in a systematic way. In this study, the logical model is developed based on the outcomes, performance and change objectives and related activities that were identified.

### 2.3. Step 3: Developing Theory-Based Methods and Practical Intervention Strategies

The formulation of the performance objectives, as well as the change objectives in Step 2 above, leads to the identification of appropriate theory-based methods and strategies. These theory-based methods are techniques and processes that are applied to achieve the intended individual and environmental changes. Theoretical methods intended to facilitate change at the individual level need to be applied differently when seeking to effect change at an ecological or environmental level. While the determinants may be the same, the techniques or delivery mode of the theoretical method may differ to achieve the intended change. Theory-based methods also inform the activities of the intervention program [32].

### 2.4. Step 4: Integrating Methods and Practical Strategies into an Intervention Workshop Program

The overall goal of step four was to design the actual intervention. This involved developing and refining the program structure and organization, as well as preparing program materials, including creating the module curriculum for the workshops.

## 3. Results

The results from this IM study are presented based on the four steps, showing how each step was implemented and practically applied.

### 3.1. Step 1: Needs Analysis

This step involved engaging the existing literature as well as 39 participants, all of whom were well-positioned to describe the challenges affecting PrEP uptake and retention amongst FSWs. Overall, the needs analysis confirmed that an intervention was necessary, as PrEP uptake is considerably low, and that there is a need for an intervention that encourages PrEP uptake and adherence, emphasizing the importance of prevention.

The barriers and facilitators of PrEP use gathered from the needs analysis are presented in Table 1, which provides a synopsis of the individual and environmental factors impacting PrEP uptake among FSWs.

### 3.2. Step 2: Identifying Objectives

The working group (described in Section 2.2) was involved in co-creating the behavioral and environmental outcomes, as well as the performance and change objectives of the intervention.

#### 3.2.1. Behavioral and Environmental Outcomes

**Figure 1:**
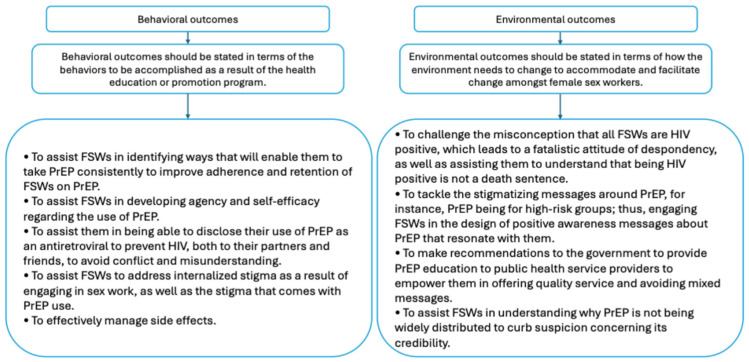
Behavioral and environmental outcomes developed by the working group.

The outcomes are presented in Figure 1 and were significant to create, as they served as the foundation for the entire intervention design process. These outcomes outlined the specific changes necessary for the intervention to be effective, focusing on what behaviors needed to be accomplished by the FSWs and how the environment needed to change to support those behaviors.

Once the behavioral and environmental outcomes were outlined, it was essential to formulate the performance objectives and change objectives matrices for each desired outcome. These matrices were key tools for IM because they provided implementers with intervention deliverables or performance objectives, enabling changes in behavior and the environment, which in turn would improve the health and quality of life of those targeted by the intervention. While the performance objectives were to clarify the actions or performances expected from intervention recipients, the change objectives specify what needs to change in the determinants (such as knowledge, attitudes, skills, self-efficacy, stigma, or social norms) for people to carry out the performance objectives. The matrices were grouped into three: (i) individual, (ii) interpersonal, and (iii) environmental.

#### 3.2.2. Matrices of Individual Change Objectives

These matrices reflect individual determinants influencing PrEP uptake and adherence among FSWs, including Self-efficacy, Agency, Hope, Future Aspirations, and Management of Side Effects. For each determinant, performance objective, and specific change objectives are outlined:

(i) Self-efficacy: The intervention program aims to build self-efficacy and confidence in PrEP use amongst FSWs. Change objectives include encouraging engagement with other PrEP-using FSWs, imaginative exercises about PrEP’s life impact, monitoring adherence progress, and developing persuasive awareness messages that foster hope, pride, and confidence.
(ii) Agency: The intervention program aims to empower FSWs to make independent decisions about PrEP. Change objectives involve assisting FSWs in defining their intentions for choosing PrEP, articulating goals and action plans, and reflecting on how their current choices align with their life vision.
(iii) Hope: The intervention program aims to foster hope within FSWs by focusing on positive aspects of their lives. Change objectives include goal setting, pathway development, encouraging gratitude journaling, and reflecting on past overcome hardships to nurture a sense of hope.
(iv) Future Aspirations: The intervention program aims to encourage FSWs to articulate their future goals. Change objectives involve shifting focus from past/present to future views, identifying desired changes and their rationale, and engaging FSWs in long-term (1, 2, 5, 10 years) time perspective exercises for their lives.
(v) Management of Side Effects: The intervention program provides practical suggestions for managing PrEP side effects. Change objectives emphasize understanding that side effects are short-lived and offering practical advice, such as taking pills at night, visiting a clinic, or speaking to a peer educator.

#### 3.2.3. Matrices of Interpersonal Change Objectives

These matrices reflect interpersonal determinants that impact PrEP uptake and adherence among FSWs, specifically addressing issues of Stigma, Partner Support, and conflict within the FSW community. For each determinant, performance objective, and specific change objectives are outlined:

(i) Stigma: The objective is to destigmatize PrEP through knowledge and positive messaging. Change objectives include educating FSWs that PrEP is an antiretroviral preventing HIV transmission, using positive messages that frame PrEP as promoting sexual agency and wellness (rather than focusing on high-risk groups), and challenging predominant negative discourses around PrEP.
(ii) Encourage support from intimate partners: The objective is to equip FSWs to negotiate PrEP use with their partners. Change objectives involve teaching FSWs how to explain PrEP’s benefits as an additional HIV prevention method and encouraging partners to also consider PrEP. Role-playing is suggested as a method for this training.
(iii) Address conflicts between FSWs on PrEP and those on antiretrovirals: The objective is to equip FSWs to handle conflict and confrontation from peers regarding PrEP legitimacy. Change objectives highlight the need to address conflict stemming from a lack of knowledge and the misconception that all FSWs are HIV positive. It also identifies underlying factors like fierce competition and jealousy within the FSW community (where being HIV-negative might be seen as an “achievement” due to high prevalence) as contributors to this conflict, suggesting dialog as a solution.

#### 3.2.4. Matrices of Environmental Change Objectives

These matrices reflect the environmental determinants crucial for the successful implementation and uptake of PrEP among FSWs. These determinants include PrEP Ambassadors, PrEP Normalization, PrEP Education, PrEP Access, and Mobility. For each determinant, performance objective, and specific change objectives are outlined:

(i) PrEP Ambassadors: The objective is to train FSW PrEP enthusiasts to educate their peers. Change objectives involve an intervention program where peer educators train interested FSWs. These trained FSWs would then volunteer as PrEP ambassadors, providing localized awareness and motivation to their friends and peers.
(ii) PrEP Normalization: The objective is to promote PrEP to the wider community to normalize its use as an HIV prevention method. Change objectives include calls for government and healthcare providers to create mass awareness messages about PrEP, targeting not only high-risk groups but also the general population. This approach aims to curb stigma and integrate PrEP as a normalized method of HIV prevention.
(iii) PrEP Education: The objective is for healthcare providers and peer educators to explain PrEP and its similarities/differences with antiretrovirals in simple language. Change objectives emphasize that FSWs need to understand the relationship between PrEP and antiretrovirals. Educators should use clear, everyday language, avoid medical jargon, and visually illustrate how the pills differ, despite their similarities.
(iv) PrEP Access: The objective is to make PrEP more widely available beyond specific NGOs, including other government clinics. Change objectives include expanding PrEP accessibility to FSWs operating outside city centers and ensuring PrEP is available at various distribution centers across South Africa.
(v) Mobility: The objective is to provide FSWs with options for managing their pill supply when traveling or away from their primary residence. Change objectives involve enabling FSWs to access PrEP in different parts of South Africa and allowing them to receive extra quantities of pills to cover periods when they are away.

#### 3.2.5. Logic Framework Depicting the Intervention Process

**Figure 2:**
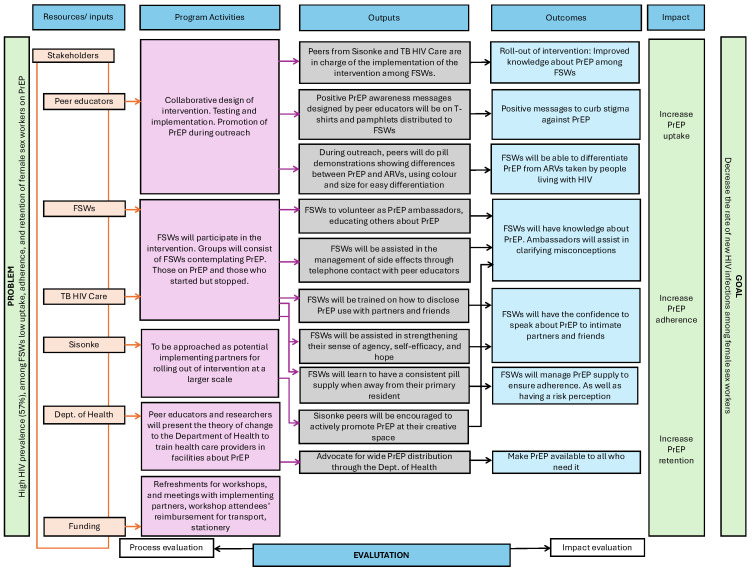
Logical model.

The logical model framework is presented in Figure 2. This framework depicts the goals, activities, inputs, and outcomes informed by the matrices of change. Furthermore, the logic framework depicts the priorities of the intervention, providing stakeholders and potential program implementers with an intervention blueprint or road map for implementation and evaluation. The model shows the logical connections between inputs (i.e., core elements and resources necessary for implementation) and outputs (i.e., the changes anticipated to result from the planned intervention) in the intervention process and the theory of change (mechanisms of change) underlying the intervention plan. The inputs include personnel (peer educators, FSWs, etc.), finances, and relevant organizations necessary to deliver the intervention. The outputs include initial/immediate products or outcomes such as improving PrEP knowledge, PrEP access, and supply.

### 3.3. Step 3: Developing Theory-Based Methods and Practical Intervention Strategies

The working group identified appropriate theory-based methods and linked them to intervention strategies relevant for achieving the performance objectives. This was accomplished by outlining all determinants and change objectives, then pairing each with suitable theories. Additionally, examples of strategies, practical applications, activities, and material needs were detailed for each objective of the determinant change. A summary of the theory-based methods is provided in Appendix A.

### 3.4. Step 4: Integrating Methods and Practical Strategies into an Intervention Workshop Program

To address the low uptake and adherence of PrEP among FSWs, the working group felt that it was important to formulate a program in the form of workshops that aligns with the change objectives established in Step 2 and the theory-based activities in Step 3. These workshops would be facilitated by peer educators among small groups of FSWs to educate them about PrEP, and to motivate and encourage the uptake of PrEP. These workshops consist of eight modules. The development and refinement of these modules were done by the working group, with the first author’s assistance, through a participatory action formative evaluation process. Through the needs analysis, the group was able to understand the context of PrEP programming, its strengths and challenges, and thus the need for the intervention. They engaged critically with the insights generated through Steps 1 to 3 of the intervention mapping, as this was the foundation for the assembly or design of the program in Step 4. The activities informed by the theory, and highlighted in Appendix A, were incorporated into the program.

A series of meetings was held during the program design phase to facilitate participatory deliberation and dialog. Formative evaluation was a cyclical process that provided multiple opportunities to modify components of the proposed intervention program to ensure fit-for-purpose and relevance, with the ultimate goal of achieving optimal outcomes. The members of the working group piloted some program components; for example, suggestions made during the planning meetings were tested to see how they would be received by FSWs during their PrEP outreach sessions. Regular feedback was provided about their respective field experiences, and amendments were made to the program where relevant. The workshop meetings with the peer educators were transformational, as they provided opportunities to share ideas, strengthen, and consolidate their approach to PrEP outreach, moving from working in silos within their respective organizations to engaging in partnerships with other organizations and stakeholders to promote PrEP.

The formative evaluation is also an opportunity to ascertain dose, consistency, usefulness, and quality of the intended intervention. Through the formative evaluation process, causal events leading to change are established and related to specific components of the intervention. Furthermore, the formative evaluation established intervention accessibility and relevance to the target audience. An example was the use of the pill differentiation method, where a peer educator demonstrated the differences between PrEP and antiretrovirals for HIV-positive people. The peer educator’s feedback indicated that this teaching method was well received, and it was thus incorporated into the program.

Another example was the use of the PrEP awareness slogans designed by the working group during the intervention mapping. These messages—Taking the power of HIV prevention with PrEP; Stay PrEPared and HIV protected with PrEP; My body, my HIV negative status is my inspiration for taking PrEP—were printed on T-shirts worn by the peer educators during outreach. The feedback was that the T-shirts sparked curiosity among FSWs and were good conversation starters about PrEP. Thus, the formative process undertaken during the intervention’s development was beneficial, as peer educators refined their ideas for the program, drawing on their field experiences.

The formative evaluation, therefore, assisted implementers in ascertaining weaknesses in the intervention design and proposed implementation process. Data collection during this process occurred before, during, and after implementation to optimize success and provide a detailed understanding of the intervention, while allowing refinements before and during the implementation phase.

Formative evaluation, furthermore, provided the implementers with an opportunity to analyze complexities that may influence the progress and effectiveness of the implementation process. Questions were asked to ascertain feasibility, context, and adaptation, as well as response towards the intervention among the target audience. This occurred during the program’s design and pretesting to guide the intervention process.

Through this formative evaluation process, the Walk in Hope PrEP workshop program was designed and consisted of two main sections. The first part focused on promoting personal growth and introspection among FSWs and was related to HIV prevention through the use of PrEP. The second part focused on promoting PrEP knowledge and stigma. These parts are described in detail in the subsequent section.

#### 3.4.1. Part 1 of the Walk in Hope PrEP Workshop Program

The first part of the program (Appendix B) was informed by the results from the needs analysis, which showed that the FSWs who adhered and were retained on PrEP, did so in part due to their personal motivations, which stemmed from self-love, having developed their own sense of agency, as well as the inspiration they received from taking care of their children. Some also had aspirations of studying further with the hope of finding other forms of employment or starting up businesses. The working group felt that the majority of healthcare interventions tended to focus on the act of taking pills daily, without regard to the human element of treatment. They also felt that FSWs have to cope with various intersecting issues such as societal stigma, discrimination, and human rights violations, which rendered them marginalized and, to an extent, dehumanized, which also contributed to a sense of apathy and a fatalistic as well as a hopeless approach to life. It was therefore important for the program to start with the process of personal development to ignite hope, encourage growth, and introspection before addressing the technical aspects of prevention. The group felt that FSWs would most likely engage effectively in preventing HIV if they had a strong sense of personal agency, purpose, and mastery over their own lives. This empowerment of the self was thus the foundation to create receptiveness for the information and motivational content of the intervention.

The process of personal development encompasses activities to promote self-examination and critical reflexivity. The program started with a tree of life activity, which is a process embedded in the narrative methodology. The purpose of the tree of life was to encourage the telling of life stories, which encouraged experiential and observational learning. It is in the understanding of a person’s life story that one can begin to see the values they hold, how they have been socialized, and what led to the problems they are experiencing. In listening intently to someone’s life story, it is then that they can be helped to create an alternative story. During the process of telling their stories, the facilitators/peer educators listened for accounts that focused on that particular FSW’s personal strengths and triumphs, as well as moments where they demonstrated personal agency. The participants were encouraged to write it down so that they could focus on the positive aspects of their personal experiences. They were then encouraged to outline their personal goals and future aspirations as well as tangible ways in which they could reach those goals to create a sense of hope. In these workshops, FSWs were assisted with cultivating self-love amidst a society that condemns them. This was done through a process of self-affirmations where FSWs were encouraged to list all the good personal qualities they possessed, as well as the different roles they occupied in society and in the lives of the people they influenced, be it their children, family members, or friends. The purpose of this exercise was to tackle internalized stigma and the shame that some women may have felt because of their engagement in sex work.

#### 3.4.2. Part 2 of the Walk in Hope PrEP Workshop Program

The second part of the program (Appendix C) was designed to encourage adherence and self-management behaviors in relation to PrEP. To achieve this, the needs analysis showed that client education is important to help individuals who take PrEP or those contemplating taking it. This was important for the purpose of adherence to the prescribed daily pill, as well as to understand the treatment process, including the related physiological side effects and other social challenges they may face in taking PrEP, such as stigma. Data from the needs analysis, including several other empirical studies of various authors [33,34,35], have shown that adherence to a prescribed treatment regimen that a patient has to take over a period of time is challenging. To engage effectively with long-term medication use, the client must make adjustments to take consistent action by changing physiological conditions and life situations. We defined this as self-management, which implies self-regulation where clients need to make decisions and take action independently of healthcare providers. The ultimate goal of self-management includes consistency in performing health behavior as well as improving the quality of the behavior, whereby the client is able to engage effectively in problem-solving and to overcome. Encouraging self-management to improve adherence was an integral part of this intervention because for PrEP to be effective in preventing HIV, it needs to be taken consistently. Evidence from the needs analysis showed that FSWs were faced with complex behavioral and environmental challenges that made engaging with this prevention method challenging. It was therefore imperative that they made adjustments to their everyday life situations so that they would be able to take PrEP consistently. These adjustments included FSWs knowing how to perceive risk when they start PrEP as well as to take PrEP consistently, managing side effects, as well as how to disclose their taking PrEP to their partners and friends, and also to manage pill supply when away from their primary residence.

#### 3.4.3. Feedback and Conclusion on the Walk in Hope PrEP Workshop Program

Once Part One and Part Two of the program have been successfully completed, participants will be invited to provide their feedback. This feedback will serve multiple purposes: we will evaluate the knowledge they have gained about PrEP through engaging and interactive games, gather their impressions and experiences with the program, including which aspects they found most beneficial or enjoyable, and solicit suggestions for how we can improve future iterations of the program to better meet their needs. This comprehensive feedback process is essential for ensuring the program remains effective, engaging, and responsive to participants’ expectations.

## 4. Discussion

As a first step of the intervention mapping process, a needs analysis was conducted with FSWs, peer educators, and a counselor to understand the barriers and facilitators of PrEP use amongst FSWs, and data from the needs analysis informed the intervention. With the assistance of a group of stakeholders, peer educators (who were also FSWs), steps 2–4 of the intervention mapping process were carried out. This culminated in a behavioral intervention program created with sex workers for sex workers. The importance of including stakeholders, such as peer educators, in this decision is supported in the literature, as it ensures that the program’s development is tailored, relevant, and acceptable to the targeted population [36,37]. Moreover, we argue that such a participatory approach would enhance effectiveness, foster trust, and ultimately promote community ownership of the intervention. Through collaboration with the peer educators, we were able to identify the primary objectives of the intervention program. These objectives were solely based on the completed needs analysis process and reflect the critical role consultation plays in translating identified needs into specific determinants and change objectives.

The formulation of matrices of change and the use of the logic model not only helped develop a systematic process for intervention development but also ensured that all stakeholders held a shared understanding of the intervention and how the program would achieve its outcomes.

The processes of an intervention mapping approach applied in this current article further reflect the significance of grounding intervention strategies in theory [24]. As such, each intervention strategy and activity, which responds to the identified determinants and change objectives, is informed by a behavioral change theory (e.g., systems change theory, information processing, and social networks and social support). In the final step, we developed a two-part Walk-in-Hope PrEP workshop program, with Part One focused on personal awareness and development, and Part Two focused on PrEP education and promotion.

Previous initiatives for STI and HIV prevention programs for FSWs have taken on top-down approaches and lacked empowerment activities, which involved sex workers [38]. Increasingly, over the years, multilevel/component interventions have moved beyond condom provision and promotion to incorporate approaches such as peer education, which has been the mainstay of HIV prevention programs amongst FSWs in different contexts [39,40]. A randomized control trial in Madagascar among 1000 FSWs found that peer condom promotion alone was not effective, but when combined with individual counseling, it reduced STIs by 30% after 6 months [41]. The Avahan study was one of the largest programs that implemented combination prevention strategies in India to reduce HIV incidence [42]. The interventions implemented were multilevel, including peer outreach, community mobilization, advocacy activities for sex workers and their clients, counseling, and clinic services. Overall results from this intervention showed a significant decline in HIV prevalence [42,43]. Evidence from the community-based SAPPH-Ire sex work program in Zimbabwe, which was concerned with ART scale-up, utilized peer-led empowerment and mobilization, which over time resulted in increased ART coverage and viral suppression among FSWs [44]. On the contrary, in the same intervention combination prevention, which included the provision of HIV testing, ART, and PrEP, did not show a significant reduction in HIV infections or viral suppression despite the uptake of services, the conclusions from this trial were that individual-level impacts do not necessarily result in population-level effectiveness [45]. This finding relates to the importance of multilevel interventions. In South Africa, the USAID PEPFAR program initiated over 1.2 million people on PrEP across seven provinces from various key and vulnerable population groups, including FSWs. Amongst other PrEP delivery techniques, this program included community-based and facility-based service delivery, supported client-centered care such as mobile clinics and community-led initiatives with peer educators to ensure the services are not only available but also acceptable to the people who need them most [46]. There was no evidence whether this large-scale PrEP program incorporated participatory methods to include FSWs or other key populations in the conception and design of these interventions. The PrEP delivery modes were also biomedically inclined to ensure sustained pill taking.

The Walk-in-Hope FSWs program aims to draw attention to the FSWs’ inner world, providing a deeper understanding of what drives FSWs to engage or refrain from health-seeking behaviors. It also seeks to educate and promote PrEP use and adherence amongst FSWs. It is an intentional program intended to create lasting behavioral changes for FSWs. The program is further aligned with humanistic approaches to HIV prevention. There have been similar HIV psychosocial interventions that have been implemented in contexts, like India, such as Sonagachi [33], termed as a risk reduction program aimed at reducing HIV infection amongst FSW. This program not only focused on HIV education and prevention but also included an empowerment component that focused on individual and group empowerment, drawing from the theory of conscientization, where sex workers are helped to develop a mentality of taking action to change their position through education and the development of self-worth and the building of hope. The impact of Sonagachi was a significant decrease in HIV infections in Calcutta as compared to other Indian cities [33]. Another risk reduction program that focused on the psychosocial well-being of FSWs as a precursor to HIV prevention was conducted in Soweto, South Africa. The peer-led creative space workshops aimed to promote conscientization and community empowerment. In these workshops, sex workers were asked to select health issues that they wanted to discuss, so the dialog was free-flowing; however, peer educators were there to create structure through facilitation. In this space, FSWs had an opportunity to connect and build solidarity, develop and nurture self-esteem and confidence, and express themselves, as well as get access to health information. FSWs who formed part of the creative space expressed that the space provided them with healing, as talking about their pain and trauma was cathartic, and the space also provided a sense of belonging. Others learnt about their human rights and were better informed on how to deal with abusive clients. A non-judgmental space with a rights-based dialog that framed sex work as work contributed to sex workers’ sense of well-being [35].

Similarly, the Walk-in-Hope program aims to contribute to PrEP uptake and adherence. The program will be carried out in two-part workshops. The focus of part one is to promote personal development, self-awareness, and hope amongst FSWs, enhanced with a sense of personal agency, self-love, and resilience. Topics covered as part of their Part One workshop will be (i) the ‘tree of life’, which is sharing of life stories and building rapport, (ii) ‘who am I? who are we?’, which is reflections on personal and collective identity and values, (iii) ‘power from within and power with others’, which would be unpacking power and power relations, (iv) ‘my actions, my responsibility’, which would focus on making difficult choices, personal responsibilities and lessons learned, (v) ‘hope’, which is fostering gratitude, and lastly (vi) ‘walking the talk, which focuses on actions that promote holistic healthcare. The second part broadly focuses on HIV prevention, what PrEP is, and PrEP stigma.

The subsequent steps to this current IM article (implementation and evaluation) are set in motion and will be realized from 2026 to 2027. The intervention will be implemented by the first author with FSW peer educators, and the evaluation will be a process and impact evaluation.

### Limitations

This study outlines the initial phases (Steps 1–4) of the Intervention Mapping framework. Consequently, the crucial stages of intervention implementation and evaluation (Steps 5 and 6) have not yet been conducted or concluded. As the program has not been enacted in a real-world context, its efficacy remains to be empirically established. Nevertheless, the detailed methodological discussion within this paper ensures the replicability of the intervention development process.

Furthermore, the generalizability of our findings is subject to two key limitations. First, participant recruitment was confined to a single province within South Africa, precluding the extrapolation of these results to other regions of the country. Second, our engagement was limited to participants from two sex work organizations in Durban. While providing valuable insights, this approach means the perspectives gathered may not fully represent the diverse experiences and views of all sex workers within the city, introducing a potential for selection bias in the recruitment process.

## 5. Conclusions

This paper demonstrates the importance of researching the types of psychosocial interventions required among key populations that can complement biomedical interventions. Many HIV interventions aimed at FSWs work with the assumption that FSWs have enough self-respect, feelings of self-worth, and will to live that motivates them to prevent HIV, without taking the time to build self-esteem that gets eroded due to stigma attached to sex work and the abuse that FSWs are exposed to. Therefore, it is important to develop interventions with FSWs that not only focus on perfunctory medical adherence but also on other humanistic aspects that inform and contribute to one’s life choices. Furthermore, it is imperative to include target populations in the design and implementation of their interventions, as they have insider knowledge of what works. Doing so is a recognition that key and vulnerable populations are human and should be seen as more than their behaviors, to understand their inner worlds.

## Figures and Tables

**Table 1:** Synopsis of personal and environmental factors affecting PrEP uptake, adherence, and retention among FSWs.

Factors	Aspects
Personal Factors	PrEP knowledge
	Agency
	Self-efficacy
	Internalized stigma
	Risk perception
	Mobility that interferes with adherence
	Inability to disclose the use of PrEP to partners and peers
	PrEP as a source of conflict between peers and partners
	Poor management of side effects
Environmental Factors	PrEP is being promoted for high-risk groups, which contributes to stigma
	Contradictory messages between the PrEP distributing organizations and healthcare workers from public health facilities
	Confusion about how PrEP differs from antiretrovirals given to people living with HIV
	Belief among female sex workers that all female sex workers are HIV positive
	Inadequate involvement of sex work organizations in PrEP distribution

## Data Availability

No data is unavailable due to privacy or ethical restrictions.

## Abbreviations

The following abbreviations are used in this manuscript:

FSWs	Female sex-workers
PrEP	Pre-exposure prophylaxis
ART	Anti-retroviral treatment

## Appendix A. Theory-Based Methods

**Table:**

Determinants and Change Objectives	Theory-Based Methods	Examples of Strategies and Practical Applications	Examples of Activities and Materials
Promote PrEP to the wider community (organizational).	Systems change (systems theory) [47].	Engage the Department of Health to discuss the benefits of widening PrEP distribution.	Have meetings with the Department of Health and present the theory of change.
PrEP access: PrEP must be accessible to the wider community.	Systems change [47].	FSWs need wider access to PrEP and not just from special clinics and NGOs. FSWs living outside the cities where the PrEP-distributing NGOs do not operate may not have access to PrEP.	Engage the Department of Health to promote and distribute PrEP at clinics and hospitals.
PrEP education: Healthcare providers and peer educators must show and explain the differences and similarities between PrEP and antiretrovirals in simple, everyday language.	Using imagery (Theories of information processing).	Educate FSWs about the similarities and differences between PrEP and antiretrovirals.	Take both the antiretroviral container and the PrEP container and show how the two containers differ and how the regimens differ. Then take out the actual pills and show the differences.
Engage FSWs in the development of awareness messages.	Framing (Protection motivation theory) [48].	Engage FSWs in the design of gain-frame/positive messages and loss-frame messages (HIV risk) about PrEP.	FSW peer educators will each brainstorm two to three messages that outline the positive aspects of PrEP use. According to the participants, the current message about PrEP is vague and says nothing specific about PrEP preventing HIV.
PrEP ambassadors	Use of lay health workers; peer education (Theories of social networks and social support) [49]. Ambassadors are change agents (diffusion of innovation theory) [50].	PrEP ambassadors will be asked to volunteer. These volunteers will come from the groups of FSWs who would have participated in the intervention program. Ambassadors will promote the use of PrEP at their brothels and streets where they work.	Peer educators will use this intervention program to train FSWs; those who are willing will be PrEP ambassadors. Ambassadors will be assisted by peer educators on verbal persuasion and will be role models for other FSWs with regard to adherence to PrEP.
Side effects management	Planning coping responses (Theories of self-regulation) [51].	FSWs will be encouraged to list possible PrEP side effects and coping mechanisms.	FSW peer educators will provide a step-by-step process on how PrEP users can cope with side effects.
Planning for access and adherence to PrEP for eventualities	Planning coping responses, self-efficacy, and self-regulation [52].	Suggestions will be provided on how to ensure a steady supply of pills when one is away from their primary residence.	FSW peer educators suggest that if FSWs know that they will be away, they need to approach the PrEP distributing organization for an extra supply of pills. They should also keep a small pill box in the bag that helps to ensure that they have some supply of their pills should they decide to stay overnight with their client.
Stigma (Destigmatize PrEP through knowledge)	Shifting perspectives (Theories of stigma and discrimination) [53].	Through PrEP education and promotion.	Using scenarios and role play to equip participants on how they can deal with stigma, as well as how they can respond to the stigma they experience from friends and partners.
Ability to disclose the use of PrEP to intimate partners	Guided practice (Social cognitive theory and theories of self-regulation) [52,54].	FSWs will be encouraged to disclose the use of PrEP to their partners.	Through scenarios and role play, FSWs will be equipped on how to disclose the use of PrEP to their partners so that they can promote trust and openness in their relationships.
Self-efficacy	Modeling (Theories of learning) [55].	FSWs need to express confidence in their ability to take PrEP and adhere to it.	FSWs will be exposed to their peers who are PrEP enthusiasts (PrEP ambassadors) and will engage with role-model scenarios.
Agency	Goal setting and self-affirmations (Goal setting theory, theories of self-regulation) [55,56].	FSWs will be encouraged to focus on the power of making decisions and taking positive action.	FSWs will engage in a reflective exercise on how they have used their agency in the past to make decisions and how they could have made better choices. They will also reflect on how having the power of choice can have an effect on their ability to prevent HIV and bring them closer to their personal goals.
Hope	Self-affirmation Goal setting (Goal setting theory, theories of self-regulation) [55,56]. Positive thinking and emotions [56,57].	FSWs will be encouraged to focus on those aspects of their lives that give them hope and meaning.	Keep a gratitude journal, as well as reflect on how they have managed to overcome adversity through resourcefulness. List all the people who benefit from their existence and ways in which they provide hope to others.
Future aspirations	Motivational interviewing Goal setting (theories of self-regulation) [58,59].	FSWs will be engaged in personal goals.	FSWs will be asked about their goals for taking PrEP. They will engage in an exercise where they will provide a visual representation of their goals and will be asked to provide a plan on how they will accomplish those goals.

## Appendix B. Program Part One: Personal Awareness and Development

Dialog 1: Tree of Life	
Activity	Objectives
1.1 Building trust among participants	1.1.1 To create a safe and supportive space to openly share feelings and experiences. 1.1.2 To build rapport among participants. 1.1.3 To explore issues of mutual respect and positive regard for each other. 1.1.4 To create an understanding that trust implies mutual respect for each other and show sensitivity to each other’s life stories.
1.2 Drawing the tree of life	1.2.1 To use the tree of life as a metaphor to tell FSWs’ life stories. 1.2.2 To draw the roots to represent their early life and childhood experiences. 1.2.3 To draw the trunk to represent their teenage life before their entry into sex work. 1.2.4 To draw the branches to represent their current life and adult experiences. 1.2.5 To draw broken branches to represent their broken dreams and painful experiences. 1.2.6 To draw leaves and fruit to represent the good aspects of their lives, hopes, and dreams.
1.3 Sharing of life stories	1.3.1 To tell their life stories—this gives FSWs an opportunity to share intimate details about themselves and their lives. 1.3.2 To assist the FSW to refocus on the positive aspect of their life story where they display positive qualities of agency, self-efficacy, hope, and resilience. 1.3.3 To list their triumphs and all the challenges they have managed to overcome. 1.3.4 To use their own past experiences of resilience and coping when faced with current difficulties.
Dialog 2: Who am I? and Who are We?	
Activity	Objectives
2.1 Reflecting on personal values, beliefs, and identity	2.1.1 To reflect on different parts of themselves that are separate from being a sex worker. 2.1.2 To engage with their multiple roles and identities. 2.1.3 To list the people in their lives who benefit from their multiple identities and roles. 2.1.4 To reflect on their goals in life. 2.1.5 To develop action plans for achieving those goals.
2.2 Clarification of values	2.2.1 To engage with their beliefs about who they are. 2.2.2 To explore and identify the values that are important to them. 2.2.3 To relate their values to everyday life and HIV prevention.
2.3 Collective identity	2.3.1 To reflect on the self in relation to the larger sex worker community. 2.3.2 To explore how they view each other. 2.3.3 To explore shared values to foster collective identity. 2.3.4 To engage with aspects related to a collective identity and responsibility, and how this relates to HIV prevention. 2.3.5 To explore the likely outcomes of a united community of FSWs to gain insight into the positive outcomes, for example, the source of support and strength to draw from.
Dialog 3: Power from within and power with others	
Activity	Objectives
3.1 Understanding power and power relations	3.1.1 To engage participants in a conversation about how they understand power and how power influences their lives. 3.1.2 To explore ways in which they deal with power relations in their intimate relationships. 3.1.3 To explore how they deal with power relations in their relationships with their peers and friends. 3.1.4 To understand their power relationships with their clients. 3.1.5 To identify ways in which they can reclaim some of their own power. 3.1.6 To explore the role of power in HIV prevention.
Dialog 4: My actions; my responsibility	
Activity	Objectives
4.1 Making difficult choices and lessons learnt	4.1.1 To identify the difficult choices they had to make in life. 4.1.2 To explore the aspects that helped them make those choices. 4.1.3 To reflect on the good and bad choices they made. 4.1.4 To identify the good choices they can make in relation to HIV prevention. 4.1.5 To foster a personal sense of responsibility to prevent HIV. 4.1.6 To explore their views about a personal responsibility to prevent HIV among their intimate partners and clients.
Dialog 5: Hope	
Activity	Objectives
5.1 Fostering gratitude and hope	5.1.1 To identify aspects that give them hope. 5.1.2 To reflect on their hopes for the future.
Dialog 6: Walking the Talk—Performativity	
Activity	Objectives
6.1 Holistic healthcare behavior	6.1.1 To develop insight into holistic healthcare (sexual, physical, spiritual, and mental health). 6.1.2 To explore barriers and facilitating factors in health-seeking behaviors that are required by an individual and by the FSW community. 6.1.3 To build skills and self-efficacy to adhere to healthy behaviors. 6.1.4 To explore strategies to encourage and keep oneself accountable in engaging in healthy lifestyles, also at a community level.

## Appendix C. Program Part Two: Pre-Exposure Prophylaxis Education and Promotion

**Table:**

Discussion Activity	Objectives
1. HIV prevention	1.1 To give participants an opportunity to explain why preventing HIV is important to them. 1.2 To engage participants in the methods they currently use to prevent HIV. 1.3 To ascertain awareness of PrEP.
2. What is PrEP?	2.1 To explore current knowledge and understandings about PrEP. 2.2 To equip participants with accurate factual information about PrEP, for example, how it works and the reasons why PrEP is referred to as an antiretroviral. 2.3 To develop an understanding of why condom use is important when using PrEP. 2.4 To explore the acceptability and accessibility of PrEP and the processes involved in initiating PrEP. 2.5 To develop insight into possible side effects of PrEP and actions to take when side effects are experienced. 2.6 To explore the differences between PrEP and post-exposure prophylaxis and appropriate use.
3. PrEP stigma	3.1 Explore facilitating factors and barriers to the use of PrEP and strategies to overcome barriers. 3.2 Gain insight into the stigma surrounding PrEP and the best ways to cope with challenges pertaining to its use. 3.3 Develop self-efficacy and skills to disclose their use of PrEP to their intimate partners and friends, as well as how to deal with negative feedback.
